# An Organic Electrochemical Transistor-Based Sensor for IgG Levels Detection of Relevance in SARS-CoV-2 Infections

**DOI:** 10.3390/bios14040207

**Published:** 2024-04-22

**Authors:** Antonio Algarín Pérez, Pablo Acedo

**Affiliations:** Electronic Technology Department, Universidad Carlos III de Madrid, 28911 Leganés, Spain; pag@ing.uc3m.es

**Keywords:** Organic Electrochemical Transistor (OECT), Immuno-Sensor, SARS-CoV-2, PEDOT:PSS

## Abstract

Organic electrochemical transistors appear as an alternative for relatively low-cost, easy-to-operate biosensors due to their intrinsic amplification. Herein, we present the fabrication, characterization, and validation of an immuno-detection system based on commercial sensors using gold electrodes where no additional surface treatment is performed on the gate electrode. The steady-state response of these sensors has been studied by analyzing different semiconductor organic channels in order to optimize the biomolecular detection process and its the application to monitoring human IgG levels due to SARS-CoV-2 infections. Detection levels of up to tens of $\mu g\;{\mathrm{mL}}^{-1}$ with sensitivities up to 13.75% [μg/mL]^−1^, concentration ranges of medical relevance in seroprevalence studies, have been achieved.

## 1. Introduction

In recent years, the quest for low-cost molecular detection and monitoring has been under study since the outbreak of the global pandemic caused by the severe acute respiratory syndrome coronavirus 2 virus (SARS-CoV-2). Current methods for the detection of this virus approved by the US Food and Drug Administration (FDA) are based on two branches. The first one is the reverse transcription-polymerase chain reaction (RT-PCR) test, based on DNA amplification due to replication by DNA polymerase as a biological machinery, and the other option is immunological-based detection tests [1,2].

The latter, antibody-based tests, are mainly based on detecting the presence of specific antibodies or antigens related to the SARS-CoV-2 virus. Antibodies play a fundamental role in the immune response, so the study of baseline levels in a person gives us an appreciable amount of information: risk of secondary infections, evaluation of the effectiveness of vaccines, immune system status, etc. [3]. In this scenario, the work presented here takes advantage of the diversity of new technologies that are emerging for the detection of biological processes of interest, specifically sensors based on organic electrochemical transistors (OECTs), to validate an immune transduction system capable of detecting antibody concentration levels related to the immune response and recovery after viral infection caused by SARS-CoV-2, with the idea to leverage the low cost, high sensitivity, and easy fabrication and integration of these devices.

Organic electrochemical transistors are used in different fields of bio-electronics, for cell monitoring in cell cultures [4,5], the monitoring of biological signals [6,7], or the detection of biomolecules [8,9], among others [10]. These transistors obtain their biosensing capabilities by coupling electrical and ionic interfaces into their architecture. Typical OECT structures consist of three terminals (gate, drain, source) and an organic semiconductor material that establishes the conduction channel between the drain and source terminals. This organic semiconductor channel is in contact with the gate terminal through an electrolyte solution. OECTs typically show operating voltages below 1 V. They have high transconductance, and, importantly, are flexible due to the use of organic channels with high Young’s moduli. All this together, along with their low manufacturing cost and biocompatibility, makes them ideal for application in molecular biosensing systems [10,11].

In this work, commercial gold electrodes AUFET30 from Metrohm Dropsens® are used. The semiconductor channel material is deposited on them, and the gate terminal, without any further surface treatment, is functionalized for the IgG-based detection of SARS-CoV-2. Different semiconductor organic materials such as Poly(3,4-ethylenedioxythiophene)-poly(styrenesulfonate) (PEDOT:PSS), graphene oxide (GO), and reduced graphene oxide (rGO) have been used for the fabrication of the sensors and characterized. Sensitivity to IgG is achieved through cysteamine/glutaraldehyde/receptor binding domain (RBD)/IgG binding complex immobilization on the surface of the base gold terminal, protecting the non-specific binding sites with bovine serum albumin (BSA) to ensure detection specificity. This type of complex is widely used in SARS-CoV-2 detection assays [12]. Several immunological studies on the dynamics of antibody levels agree that IgG-antiRBD and IgG-antiSpike are indicative for assessing these dynamics during the first three months after SARS-CoV-2 infection in both saliva and serum [13,14,15,16,17,18], reaching mean IgG concentration levels of 25.5 ± 47.7 $\;\mu g\;{\mathrm{mL}}^{-1}$ in SARS-CoV-2 affected patients, and studies also detail concentration levels ranging from 1 to 100 $\mu g\;{\mathrm{mL}}^{-1}$ [19].

The sensors fabricated and tested in this work show a linear measurement range of human IgG concentrations from 5 $\mu g\;{\mathrm{mL}}^{-1}$ to 30 $\mu g\;{\mathrm{mL}}^{-1}$, making them usable in human-IgG monitoring systems in people exposed to SARS-CoV-2 and those vaccinated, to determine the effectiveness of vaccines and to be used in seroprevalence studies to estimate the proportion of the population that has been previously infected or vaccinated against SARS-CoV-2. This kind of data is crucial to understand the spread of the virus in a community, to analyze the infection rate, and to assess the immunity rate of the population, fundamental tools to guide public health strategies.

## 2. Device Description

### 2.1. Organic Electrochemical Transistors (OECTs)

Organic Electrochemical Transistors (OECTs) are derivatives of the organic field-effect transistor (FET), whose special feature is that they do not have an oxide passivation layer between the gate terminal and the semiconductor channel, but the gate and the channel are in contact through an electrolyte solution [20]. This work makes use of this kind of structure.

### 2.2. Operating Principle

The principle behind drain current modulation in OECTs relies on the control of the electric field applied to the semiconductor channel (due to the application of a gate potential $V_{G}$), which induces doping/de-doping processes in the organic semiconductor due to the ionic injection of charge carriers from the electrolytic medium into the transistor channel [11]. This process is mediated by the capacitance and resistance of the medium, and the applied potential difference [20,21,22].

The drain current ($I_{D}$) of these transistors will vary depending on the bias point at which the transistor is biased. This bias point establishes a level of base doping, and therefore conduction, in the organic semiconductor channel, presenting different dynamics in the drain current depending on the doped regions [23]. In our application, we bias the device in the region with the highest transconductance $g_{m}=\frac{\partial I_{ds}}{\partial V_{gs}}$ and the highest current level. This operating point occurs in the saturation region of the device, where the current remains constant in the face of changes in the drain potential $V_{D}$, reaching a value that only varies as a function of $V_{G}$. In some applications, higher $V_{G}$ levels are needed to bias the transistor in the saturation region due to the capacitive divider formed between the gate capacitance and the channel capacitance [24], so the two regions we will be working in are described as follows [20]:

(1) $$I_{D,sat}=-g_{m}\frac{V_{D,sat}^{2}}{2V_{p}}\;\;V_{D}\leq V_{D,sat}$$

(2) $$I_{D}=g_{m}\left[1-\frac{V_{G}-V_{D}/2}{V_{p}}\right]V_{D}\;\;V_{D}\leq V_{G}$$

(3) $$\begin{array}{c} V_{D,sat}=V_{G}-V_{p}\;\;g_{m}=q\mu p_{0}T\frac{W}{L} \end{array}$$

where $g_{m}$ is the transconductance of the transistor, *q* is the electron charge, μ is the hole mobility, $p_{0}$ is the initial hole density, *T* is the temperature, $\frac{W}{L}$ is the aspect ratio, and $V_{p}$ is the pinch-off voltage, which determines the saturation of the device similarly to depletion FETs.

### 2.3. Sensing Principle

The detection principle behind these transistors is the potentiometric translation at the gate electrode–electrolyte interface. The potential at the interface varies due to molecular bonding, which causes a variation in the double layer capacitance and the effective gate voltage ($V_{G,eff}$) [9,10]. This gate voltage has two components, a term dependent on the gate and channel capacitances ($V_{G_{0}}$), and a variable term that depends on the analyte concentration ($V_{sen}$), which is given in terms of the intrinsic charge of the junction complex formed as we can see in Equations (4) and (5) [25,26,27]:

(4) $$V_{G,eff}=V_{G_{0}}+V_{sen}$$

(5) $$\begin{array}{c} V_{G_{0}}=\frac{1}{1+\frac{c_{c}A_{c}}{c_{g}A_{g}}}V_{G}\;\;V_{sen}=t_{M}\frac{nQ_{M}}{\epsilon_{0}\epsilon_{r}} \end{array}$$

where $c_{x}A_{x}$ represent the the product area times the surface area capacity for the gate (*_g_*) and the channel (*_c_*), *n* is the density of molecules on the electrode surface (related to the molecular concentration), $Q_{M}$ is the pure charge of a molecule, $\epsilon_{r}$ is the relative dielectric constant of the molecular layer, $\epsilon_{0}$ is the free space dielectric permittivity, and $t_{M}$ is the effective interaction thickness of the molecular layer.

**Figure 1:**
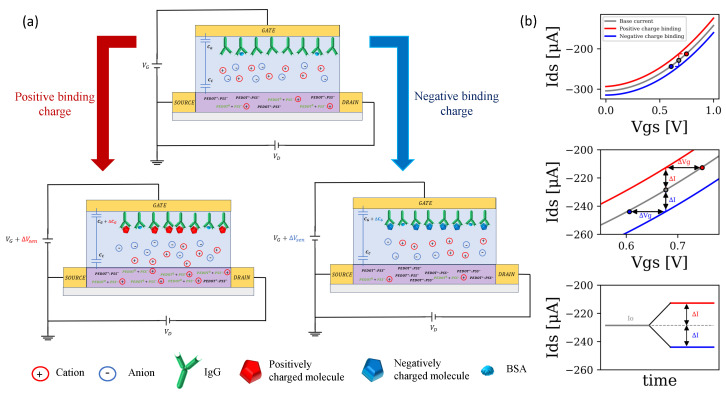
(**a**) Process of modifying the internal structure of an OECT based on an immune-binding complex detection with a PEDOT:PSS channel. The detection of molecules with different types of charge is represented. (**b**) Variation of device drain currents due to molecules detection.

The detection scheme for the case of a PEDOT:PSS channel OECT based on an immune-binding complex detection is presented in Figure 1 for positively (red) and negatively (blue) charged molecules in the case of the organic semiconducting polymer PEDOT:PSS (the one that is used in this work). The intrinsic doping and de-doping reaction of the material will be discussed in detailed in Section 3.3.1.

Figure 1a shows how molecular adhesion at the gate interface induces a shift in the potential $V_{G,eff}$ and effective double layer capacitance ($C_{g}$ gate capacitance), resulting in a variation of the base drain current of the device represented in Figure 1b. This variation of current with respect to the base current (zero concentration) will be the figure of merit (FOM) to quantify the changes in analyte concentration at the gate, as is typically done in the literature [28,29,30]. See Equation (6):

(6) $$FOM=|\frac{I-I_{0}}{I_{0}}\left|=\right|\frac{\Delta I}{I_{0}}|$$

This parameter has been used as a figure of merit in many studies and can be seen as the relative displacement in terms of current with respect to the base current, which we will always treat as an absolute value.

## 3. Materials and Methods

### 3.1. Reagents and Chemicals

Almost all experimental reagents were purchased from Sigma-Aldrich (Madrid, Spain), including poly(3,4-ethylenedioxythiophene)-poly(styrenesulfonate) in its high conductivity form, graphene oxide (GO), sodium borohydride (NaBH_4_) for the reduction of graphene oxide (rGO), phosphate-buffered solution (PBS), bovine serum albumin (BSA), deionized (DI) water (18.6 MΩ), cysteamine, Syndrome coronavirus-2 receptor-binding domain (SARS-CoV-2-RBD), glutaraldehyde, and human immunoglobulin G (H-IgG). Chistosan was purchased from Cymit Química S.L. (Barcelona, Spain).

### 3.2. Characterization Set-Up

The characterization of the I–V curves of our sensor were carried out using a Rigol DP832 power supply, a Keithley 6487 picoammeter, and a Keysigth Truevolt 34461A for potential application, gate current ($I_{G}$) monitoring, and drain current ($I_{D}$) monitoring, respectively. The system was controlled by SCPI commands using Python 3.8.3 as the working environment for instrumentation control.

For the transistor characterization, the voltage sweep was performed at a rate of $30\;\mathrm{mV}\cdot s^{-1}$ for the drain terminal and $10\;\mathrm{mV}\cdot s^{-1}$ for the gate terminal, while maintaining a 0.1 M electrolyte PBS solution. For the validation of the sensor for the detection of H-IgG, 30 min of incubation were given for each of the samples analyzed, during which at least three electrical characterizations of the characteristic curves of the device were subsequently obtained, proceeding in an analogous manner to the initial characterization.

### 3.3. Fabrication of the OECTs

The organic electrochemical transistors were fabricated on commercial AUFET30 electrode systems with 4 gold (Au) electrodes: gate, drain, source, and bulk, purchased from Metrohm DropSens® (Oviedo, Spain). These systems feature a coplanar design of a series of Au electrodes on a plastic substrate of dimensions $L34\;\times \;W10\;\times \;H0.175\;{\mathrm{mm}}^{3}$. The semiconductor channel area is interdigitated with six bands between the source and drain electrode with a spacing of 30 μm and a band length of 270 μm. The gate electrode has a rectangular structure with an area of $9\;{\mathrm{mm}}^{2}$ at a distance of 2 mm from the channel. The BIDSCFET connector from Metrohm Dropsens® (Oviedo, Spain) was used to access the AUFET30 terminals.

For the fabrication of the organic transistors, three types of organic semiconductor materials have been studied: PEDOT:PSS, graphene oxide (GO), and reduced graphene oxide (rGO).

#### 3.3.1. PEDOT:PSS

**Figure 2:**
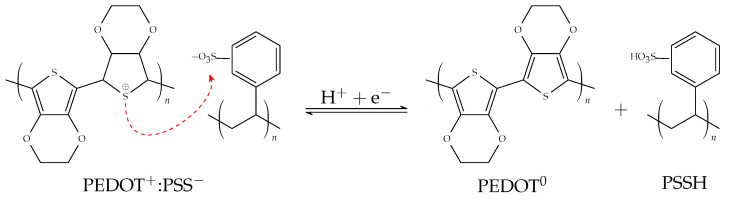
Redox reaction in the doping and de-doping process of the PEDOT:PSS material. On the left is the natural doped and conductive form. On the right is the de-doped and non-conductive form. The stabilisation of the positive charge of PEDOT with the deprotonated PSS, which acts as a negative counterion and p-type dopant, is represented by the red arrow.

Poly(3,4-ethylenedioxythiophene)-poly(styrenesulfonate) or PEDOT:PSS is a p-doped semiconductor material with high biocompatibility and high thermal, electrical, and electrochemical stability. It is composed of a polymer blend of polytheophene doped with PSS anions that are added to the PEDOT compound to compensate for the shortage of negative charges. This makes PEDOT:PSS a conducting compound in its natural state (by doping PEDOT with PSS) [26,31,32]. The ionic fusion in the channel is responsible for the variation of the conductivity of the OECTs. In the case of PEDOT:PSS, it is based on the doping/de-doping of PEDOT from its conducting state ${\mathrm{PEDOT}}^{+}$ to its non-conducting state ${\mathrm{PEDOT}}^{0}$ following the migration of cations from the electrolyte medium to the PEDOT:PSS film, driven by the variation in the potential $V_{G}$ applied on the gate electrode, as can be seen in the reaction shown in Equation (7). With the application of a positive potential at the gate electrode, an accumulation of anions will be induced in this region, while the cations of the electrolyte penetrate into the polymer channel compensating for the free sulphonate anions of the PSS. This leads to a doping removal from the PEDOT, and this causes the absolute value of the drain current to decrease due to a depletion in the charge carriers as can be seen in Figure 2 [22,26,31].

(7) $$PEDOT^{+}:PSS^{-}+H^{+}+e^{-}⇌PEDOT^{0}+PSS^{-}:H^{+}$$

As mentioned above, PEDOT:PSS in its high conductivity form obtained from Sigma-Aldrich (Madrid, Spain) was used. Chitosan was also used to stabilize the channel at a concentration of 0.1 M. For the creation of the semiconductor channel, 1 μL of the PEDOT:PSS solution was drop-cast and allowed to dry at room temperature for 20 min. After drying, 1 μL of 0.1 M chitosan was added to the PEDOT:PSS channel and allowed to dry at room temperature for another 20 min.

#### 3.3.2. Derivatives of Graphene

The use of graphene-derived materials, such as graphene oxide (GO) and reduced graphene oxide (rGO), is very popular for the fabrication of bio-compatible devices due to their excellent properties [33], being fundamental for the creation of scaffolds in tissue engineering [34]. Based on their wide use in bio-molecular sensing applications [35,36], we decided to characterize its electrical behaviour as a semiconductor channel. In single-layer graphene, the conductivity is mainly determined by the transport of charge carriers within the basal plane of the carbon. GO reduction essentially focuses on the removal of these functional groups from the basal plane [37].

In the case of graphene oxide (GO), we proceeded analogously to the fabrication of PEDOT:PSS channels. An amount of 1 μL of the compound was deposited and allowed to dry at room temperature for 20 min. The channel was then stabilized with 1 μL of 0.1 M chitosan and allowed to dry for another 20 min.

To obtain reduced graphene oxide (rGO), the commercial graphene oxide compound purchased from Sigma-Aldrich (USA) was reduced with sodium borohydride (NaBH_4_) like the procedure shown in [33]. An amount of 1 μL of the compound was deposited and allowed to dry at room temperature for 20 min. The channel was then stabilized with 1 μL of 0.1 M chitosan and allowed to dry for another 20 min.

### 3.4. Surface Functionalization

**Figure 3:**
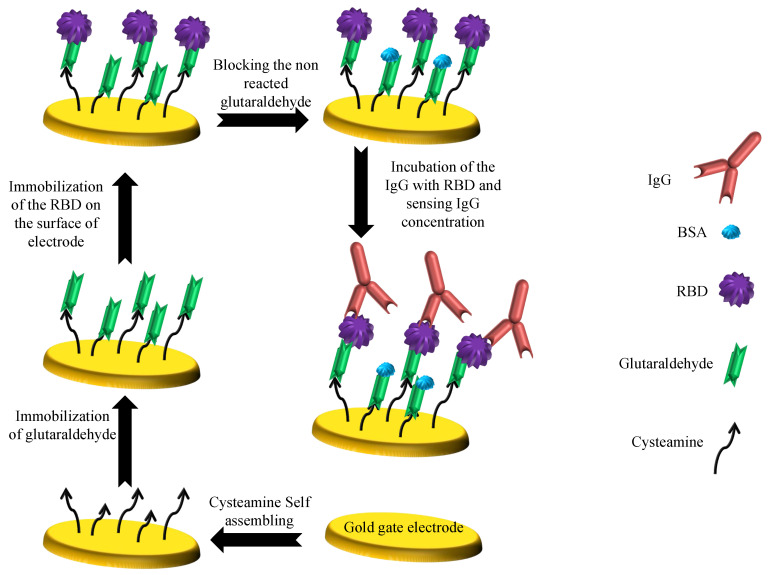
Scheme of the process of surface functionalization of the OECT-biosensor for human IgG detection. Adapted from [12].

To detect human IgG antibodies, we must immobilize the receptor binding domain on the gold surface of our gate terminal. To do this, we will form the cysteamine/glutaraldehyde/RBD/IgG complex for detection as described in the literature [12]. Figure 3 shows the process of surface functionalization.

For this functionalization, cysteamine was self-assembled on the surface of the gold gate electrode using 50 μL of 0.01 M aqueous cysteamine solution by drop-casting onto the gold gate electrode, followed by four hours of incubation time at room temperature. After that, the gold gate electrode was rinsed generously with PBS and 0.1 M DI (deionized water) and dried at room temperature. To attach the SARS-CoV-2 receptor binding domain (antigen) to the gate surface, the amino groups of the self-assembled cysteamines interacted with glutaraldehyde. To do that, a 2.5% glutaraldehyde solution in PBS (0.1 M, pH 7.4) was dropped onto the surface of the cysteamine-functionalized gold gate and allowed to interact with amine groups for 60 min. The glutaraldehyde-activated electrode was thoroughly rinsed with PBS and incubated with 50 μL of 10 $\mu g\;{\mathrm{mL}}^{-1}$ RBD of SARS-CoV-2 (antigen) for 4 h. Finally, the gold gate electrode was blocked with 50 μL of 0.5% bovine serum albumin (BSA) in 10 mM PBS on the surface of the gold gate electrode at room temperature for 15 min to prevent possible non-specific binding of glutaraldehyde molecules to IgG (Figure 3).

## 4. Results

### 4.1. Characterization of the Static Response of the OECTs

**Figure 4:**
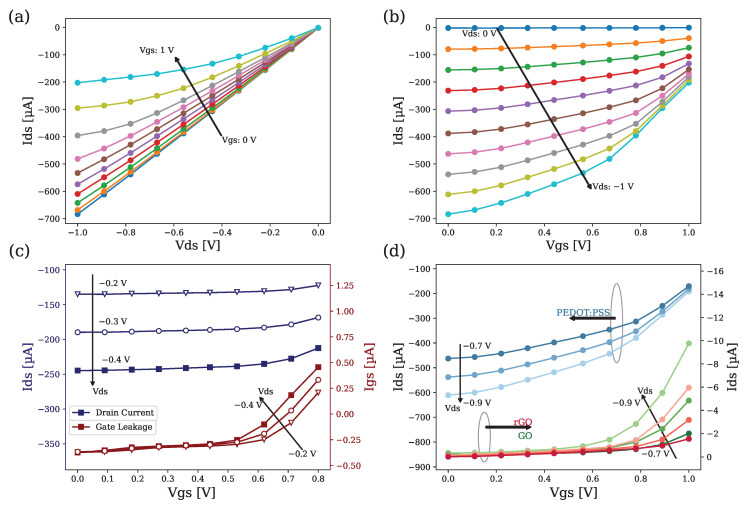
(**a**) Transistor output characteristic curve ($I_{D}$ vs. $V_{D}$). (**b**) Transistor transfer characteristic curve ($I_{D}$ vs. $V_{G}$) for PEDOT:PSS OECTs. (**c**) ($I_{D}$ vs. $V_{G}$) on the blue axis and ($I_{G}$ vs. $V_{G}$) on the red axis for PEDOT:PSS OECTs. (**d**) ($I_{D}$ vs. $V_{G}$) for the PEDOT:PSS channel on the blue (left axis) and ($I_{D}$ vs. $V_{G}$) for the GO and rGO channels (right axis) for different $V_{D}$ sweeps.

Figure 4 shows the characteristic curves of the organic electrochemical transistors for the different organic channels used. We can see how the transconductance $g_{m}=\frac{\partial I_{ds}}{\partial V_{gs}}$ of the devices (the slope of the transfer characteristic curve), which translates into an efficiency in the transduction of the device, increases with $V_{G}$ according to the model of [20].

Figure 4a,b (I–V and transfer characteristics of PEDOT:PSS channel OECTs) show that, as $V_{G}$ increases, the channel shrinks due to the channel doping process, increasing the impedance of the organic semiconductor and therefore decreasing the drain current due to the de-doped effects induced by the $V_{G}$ increase. We can also see how the current saturation effect only occurs for the case of high gate potentials, because the $A_{c}$/$A_{g}$ ratio dominates in the structure as detailed in [24].

Figure 4c shows that, if we increase $V_{G}$ and approach voltages of more than 0.7 V, $I_{G}$ tends to increase exponentially with respect to its initial value [38]. This effect is due to the non-linear relationship between current density and voltage excursions around the electrode equilibrium point, which means that, as $V_{G}$ increases, the current density at the gate electrode also increases; therefore, the $I_{G}$ levels reached causes the surface gold that forms the gate of the OECT to be removed and damage the device. Additionally, this effect is intensified by $V_{D}$. Because of this, the voltage range for the study was limited to $0\leq V_{G}\leq 0.6$ V and −0.4 V $\leq V_{D}\leq 0$.

Figure 4d shows that the currents provided by the PEDOT:PSS transistor are two orders of magnitude higher than those provided by the graphene derivatives OECTs (around nA as reported in [35]), so the choice of using PEDOT:PSS as a semiconductor material in the development of the final sensors is reinforced.

### 4.2. Immuno-Sensing

To extract the sensitivity of the device for the detection of H-IgG, different solutions with concentrations ranging from 5 $\mu g\;{\mathrm{mL}}^{-1}$ to 60 $\mu g\;{\mathrm{mL}}^{-1}$ were applied to the gate of the device functionalized with the binding complex described in Section 3.4. The characterization process described in Section 3.2 was used to analyze the variation of the device current as a function of increasing H-IgG concentration.

**Figure 5:**
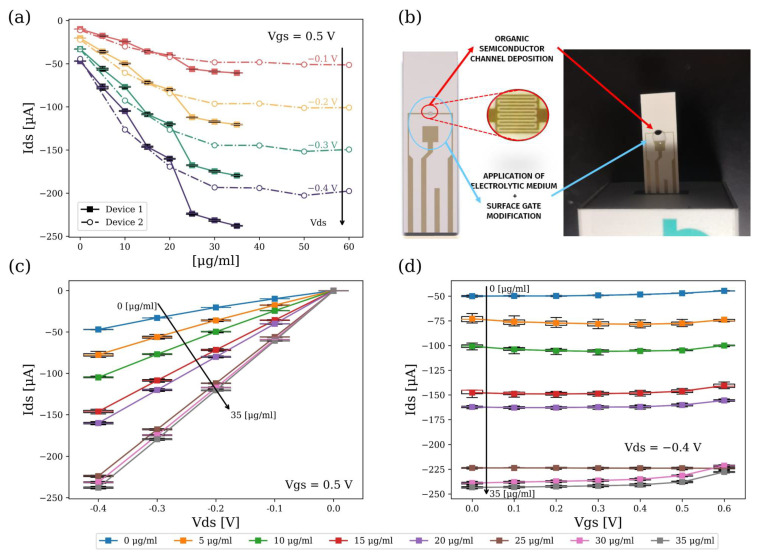
(**a**) Data from two devices ($I_{D}$ vs. H−IgG in [μg/mL]) for different $V_{D}$ values, where $V_{G}$ = 0.5 V. (**b**) OECT employed and an image of the sensing area. (**c**) Output characteristic curve ($I_{D}$ vs. $V_{D}$) for different H-IgG concentrations, where $V_{G}$ = 0.5 V. (**d**) Transfer characteristic curve ($I_{D}$ vs. $V_{G}$) for different H-IgG concentrations, where $V_{D}$ = −0.4 V.

Figure 5a shows the response of the drain current as a function of the H-IgG concentration in $\mu g\;{\mathrm{mL}}^{-1}$ of two devices fabricated (Device 1 and 2) at different $V_{D}$ values while maintaining a $V_{G}$ of 0.5 V. Figure 5b shows the actual sensors used. We can see the four Au electrodes that make up the structure of our OECT sensor. The interdigitated area highlighted in red is formed by equispaced Au bands and forms the space where the PEDOT:PSS channel is deposited.

Figure 5c,d show the output and transfer characteristic curves of the sensor for variations in H-IgG concentration at $V_{G}$ = 0.5 V, and at $V_{D}$= −0.4 V, respectively. We can see that, as the concentration increases, the drain current increases in absolute terms due to the increase of charge on the gate electrode caused by the binding of the antibodies to the RBD domain (see Equations (4) and (5)).

The results shown in Figure 5c,d sustain this explanation. There is a decrease in the effective gate voltage $V_{G,eff}$ (i.e, we can see that, at the same gate voltage $V_{G}$, we obtain higher drain currents $I_{D}$ (see Figure 1)), because H-IgG binding at the gate electrode junction complex causes a charge to build up at the interface and establishes a lower cation injection rate into the channel, increasing the conductivity of the PEDOT:PSS. Note the relationship between Figure 4a and Figure 5c, which highlights this behaviour, as we see how the increase in concentration in the output characteristic curve has the same effect as a reduction in $V_{G}$.

Figure 5a also provides information about sensor saturation and inter-device variability. We observe that device saturation is reached at around 35 $\mu g\;{\mathrm{mL}}^{-1}$, which is when $V_{G}$ reaches the point that causes the maximum current level for a given $V_{D}$ potential, shifting the reaction shown in Equation (7) towards the reactants.

**Figure 6:**
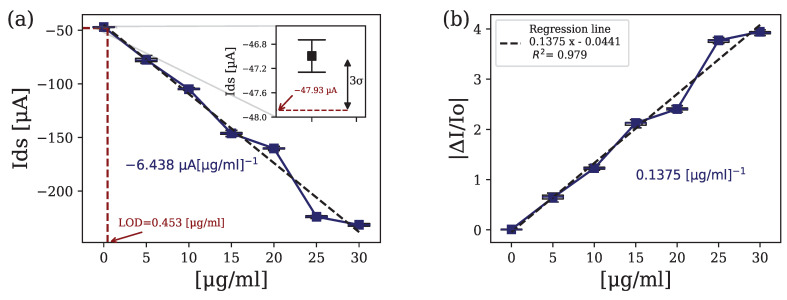
(**a**) ($I_{D}$ vs. H−IgG in [μg/mL]) and limit-of-detection (LOD) extraction based on 3-σ estimations. (**b**) ($\Delta I/I_{0}$ vs. H-IgG in[μg/mL]) and regression line fitted to the data for $V_{G}$ = 0.5 V and $V_{D}$ = −0.4 V.

We also observe that, despite sharing the same environmental conditions in the fabrication process of the devices (to avoid any variability), the response of the two devices shown in Figure 5a is different above 20 $\mu g\;{\mathrm{mL}}^{-1}$ concentration. The dynamics and current levels at low concentrations are identical (with an almost linear behaviour), but the currents near saturation vary because these devices are very sensitive to the process of deposition and fixation of the organic channel and are strongly conditioned by the adhesion of the organic channel to the gold substrate. The identified linear region is further studied in Figure 6.

Figure 6 represents the static response of the device under variations in the concentration of H-IgG (calibration curve) for $V_{G}$ = 0.5 V and $V_{D}$ = −0.4 V. The obtained linear regression for the data is also shown. From this linear regression, we obtain a sensitivity in terms of a current of $-6.438\;\mu A{\left[\mu g/\mathrm{mL}\right]}^{-1}$. The regression line, with respect to the baseline current (PBS), is y = −0.0441 + 0.1375x, where x is the analyte concentration in $\mu g\;{\mathrm{mL}}^{-1}$, and y is the figure of merit as defined in Equation (6). We can also obtain from this data the detection limit for the sensors analyzing the standard deviation of the base set of samples (zero concentration) according to 3-σ estimations reaching a limit of detection (LOD) of $453\;\mathrm{ng}\;{\mathrm{mL}}^{-1}$.

The measurements presented have been taken a minimum of three times to analyze the deviation and the repeatability of the data, and to ensure the correct behaviour of the device in terms of intra-device variation.

## 5. Conclusions

This work demonstrates the transduction capability of PEDOT:PSS-based OECT sensors for the detection of human IgG antibodies, showing, in the best case, sensitivities of $13.75\%{\left[\mu g/\mathrm{mL}\right]}^{-1}$ with detection limits of $453\;\mathrm{ng}\;{\mathrm{mL}}^{-1}$ and currents down to 0.25 mA in a linear detection range up to $30\;\mu g\;{\mathrm{mL}}^{-1}$.

Different organic materials have been studied to evaluate their performance, and we have shown that channels based on PEDOT:PSS present several advantages for these organic electrochemical transistors. PEDOT:PSS-based OECTs provided currents in the order of magnitude of hundreds of μA, with well-defined characteristic curves. They have also been widely studied, and in the literature, there are physical and electrochemical models that help in the understanding of the transduction process [11,20]. In contrast, we have shown that graphene-derived channels provide with current magnitudes two orders of magnitude smaller and have poor transfer characteristic curves for application in these organic transistors, as was already reported in [35].

The affordable fabrication of these sensors based on commercial gold electrode architectures, along with the low operation voltages and easy read-out with simple electronics, makes possible the integration of these sensors into compact, low-cost, transduction systems, with a potential major impact in the field of biosenging [10]. However, further research is needed to solve the variability obtained in the response of the different devices that could be overcome through fabrication process optimization.

We must emphasize, as one of the main novelties of this work, that previous studies on OECTs for IgG detection are oriented towards early and very selective IgG detection with detection ranges that do not exceed hundreds of $\mathrm{pg}\;{\mathrm{mL}}^{-1}$ in the vast majority of cases [27,39]. The results presented here demonstrate that we were able to monitor H-IgG antibody concentration levels with medical relevance, as those are closely correlated to the actual immune response after SARs-CoV-2 infection in serum [19]. In this sense, our sensors could be useful for seroprevalence studies and vaccine dynamics studies, helping to measure prolonged H-IgG levels over time, which are crucial for understanding viral infections in communities.

## Data Availability

Data are contained within the article.

## Abbreviations

The following abbreviations are used in this manuscript:

LD	linear dichroism
OECT	organic electrochemical transistor
H-IgG	human immunoglobulin G
FDA	US Food and Drug Administration
RT-PCR	real-time reverse-transcription polymerase chain reaction
GO	graphene oxide
rGO	reduced graphene oxide
BSA	bovine serum albumin
FET	field effect transistor
PEDOT:PSS	poly(3,4-ethylenedioxythiophene)-poly(styrenesulfonate)
PBS	phosphate buffered saline
DI	deionized water
SCPI	Standard Commands for Programmable Instruments
RBD	receptor binding domain
LOD	detection limit
